# Supplementary material for: Long Non-Coding RNA H19 Prevents Lens Fibrosis through Maintaining Lens Epithelial Cell Phenotypes
Source: Cells. 2022 Aug 17;11(16):2559. doi: 10.3390/cells11162559 (PMC9406623; doi:10.3390/cells11162559)
Supplement: Supplementary file 1 [file cells-11-02559-s001.zip › cells-1835889-supplementary.pdf]

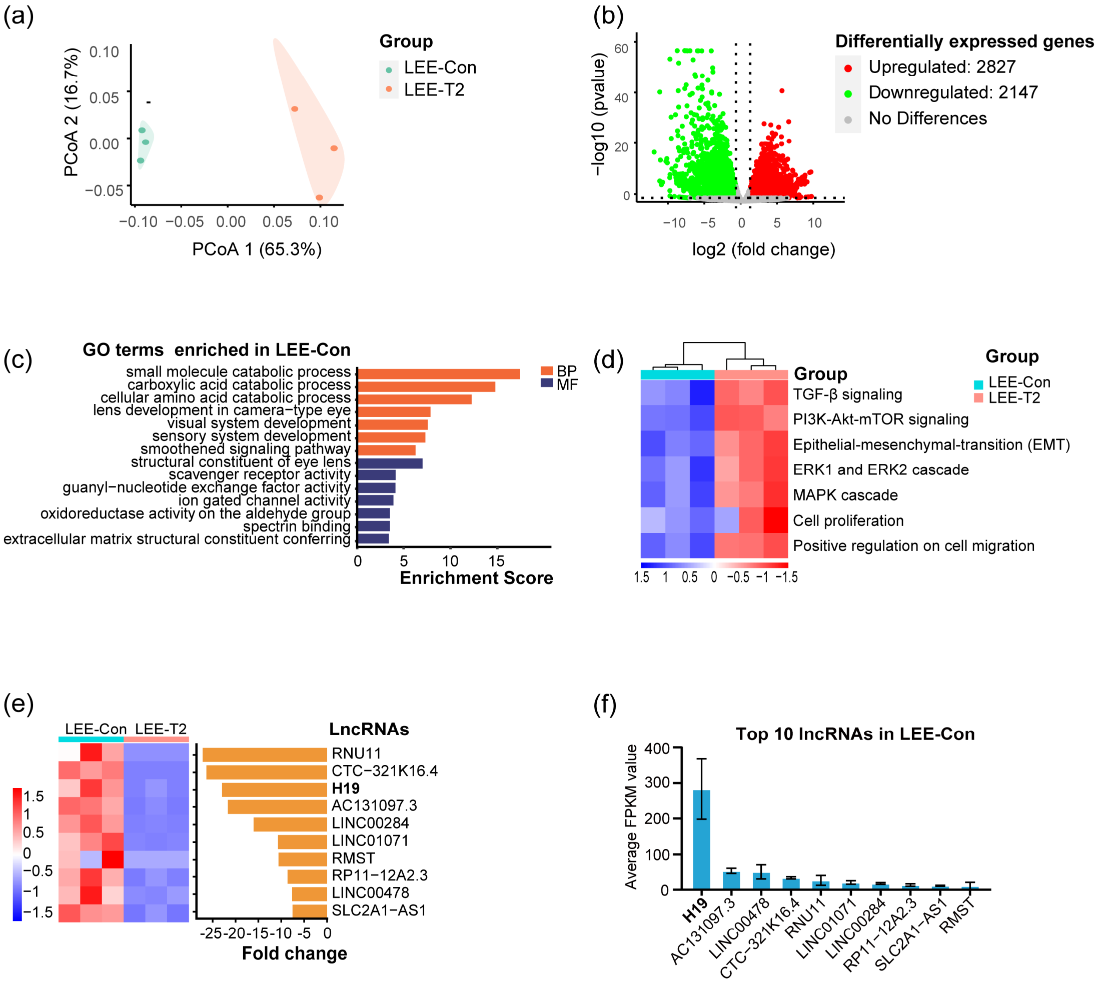

Figure S1. *H19* was identified by RNA-sequencing in human lens epithelial explants by exposure to TGF-β2. (a) Principal Co-ordinates Analysis (PCoA) of LEE-Con and LEE-T2. (b) Hierarchical cluster analysis generated a heatmap to reveal differentially expressed genes (DEGs). Red indicated upregulation, while green indicated downregulation. (c) Gene Ontology (GO) terms enriched by downregulated genes in LEEs by exposure to TGF-β2. BP, biological processes; MF, molecular functions. (d) GO terms activated by TGF-β2 induction. (e) The top 10 downregulated long non-coding RNAs (LncRNAs) were ranked according to their fold changes. (f) The top 10 downregulated lncRNAs were sorted based on their expression, among which *H19* ranked on top with the greatest FPKM values. LEE, lens epithelial explant.

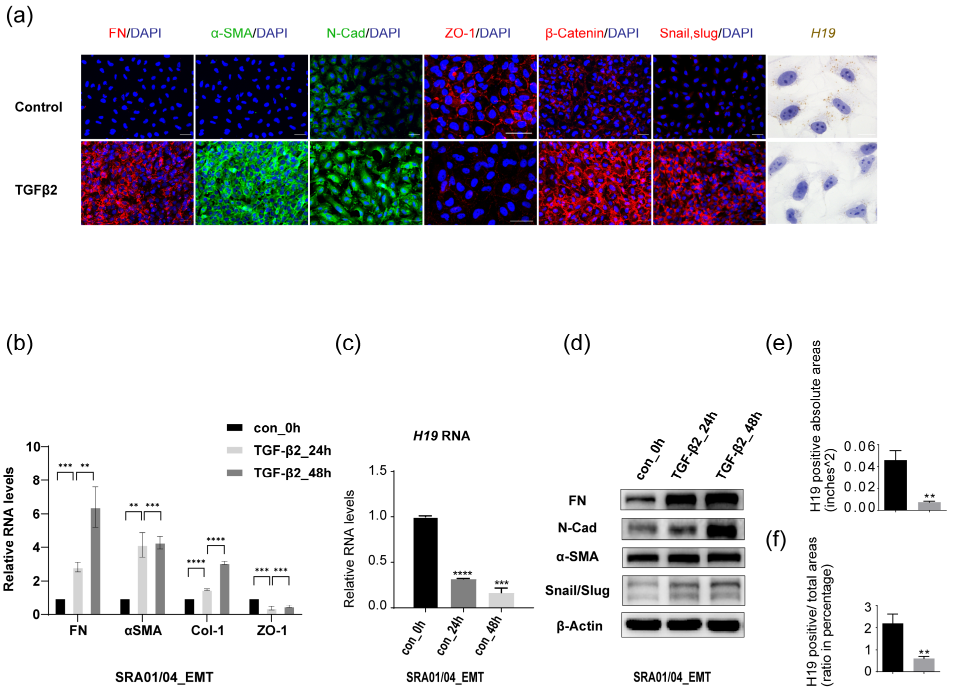

Figure S2. Duration-dependent analysis of *H19* expression in TGFβ2-treated SRA01/04 cells.

(a) Immunofluorescent staining of mesenchymal markers (FN, α-SMA, and N-Cad), epithelial markers (ZO-1, β-catenin), and Snail/Slug. Scale bars, 50 μm. *H19* probes were specifically hybridized to *H19* expressed within SRA01/04 cells exposed to TGF-β2 (5 ng/mL, 48 h), in comparison to control group. Scale bars, 20 μm. (b) RNA analysis of mesenchymal markers, including FN, α-SMA, COL1A2, and epithelial marker ZO-1, in SRA01/04 cells without or with TGF-β2 (5 ng/mL, 24h and 48h, respectively), using β-actin as the internal control, normalized to control group (con_0h). (c) *H19* RNA expression in SRA01/04 cells in response to TGF-β2 was measured using quantitative real-time PCR. (d) Western blots detected expression changes of mesenchymal markers (FN, α-SMA, N-Cad) and key transcription factors (Snail and Slug). Experiments were repeated separately for least 6 times and representative western blots were shown. (e-f) Quantification of the absolute value of *H19* positive areas (e) and the ratio of *H19* positive area to total area within each cell (f). All data were all shown as mean ± SD. ** p<0.01, *** p<0.001, **** p<0.0001 vs control group.

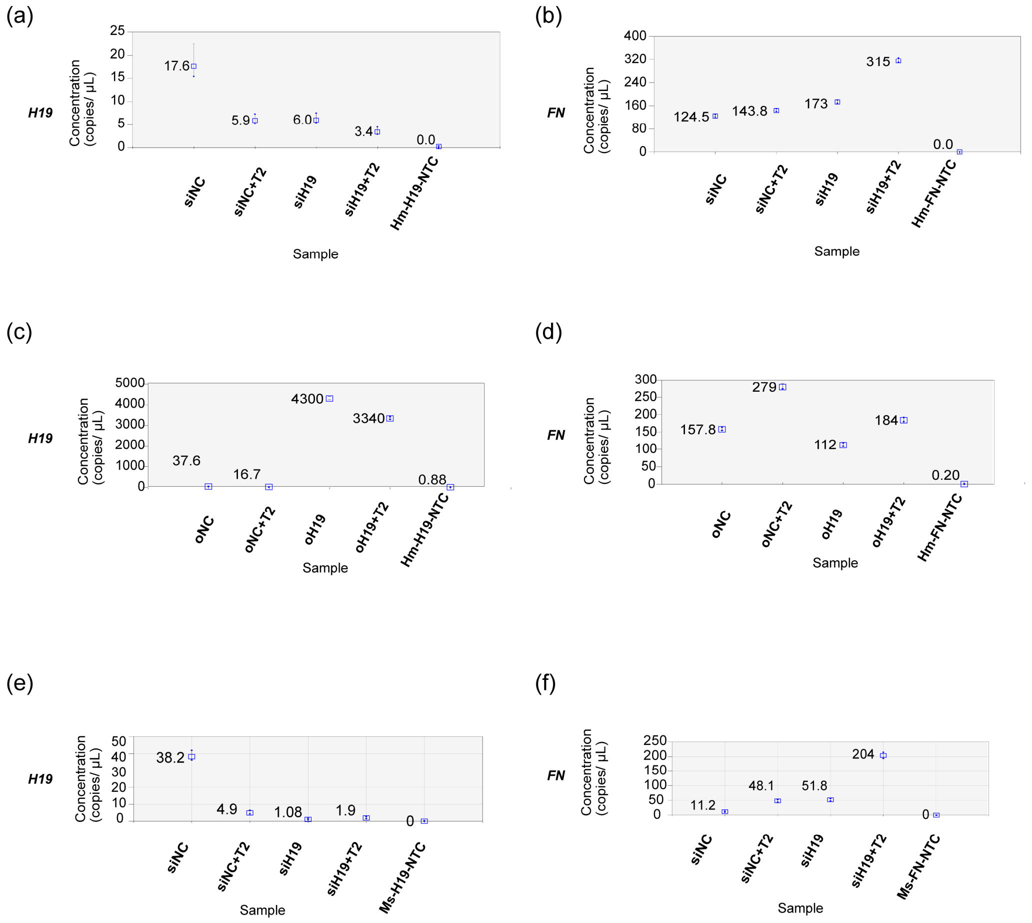

Figure S3. Digital droplet PCR validated the function of *H19* during TGF-β2-induced EMT in both human lens epithelial explants and mouse whole lens culture.

(a-b) *H19* expression and FN mRNA levels (copies/µL) were detected in human lens epithelial explants by digital droplet PCR after transfection with si*H19*-002 for 24 hours and further exposure to TGF-β2 for 24 hours. (c-d) After infection with *H19*-overexpressed lentiviruses for 24 h and further exposure to TGF-β2 for another 24 h, *H19* expression and FN mRNA levels (copies/µL) were detected in human lens epithelial explants by ddPCR (digital droplet PCR). (e-f) *H19* expression and FN mRNA levels in mouse lens epithelial cells were detected by ddPCR (digital droplet PCR) after the mouse whole lens explants were cultured with transfection agents for 24 hours and further exposed to TGF-β2 for 24 hours. Graph demonstrated absolute RNA concentration (copies/µL). NTC, no template control.

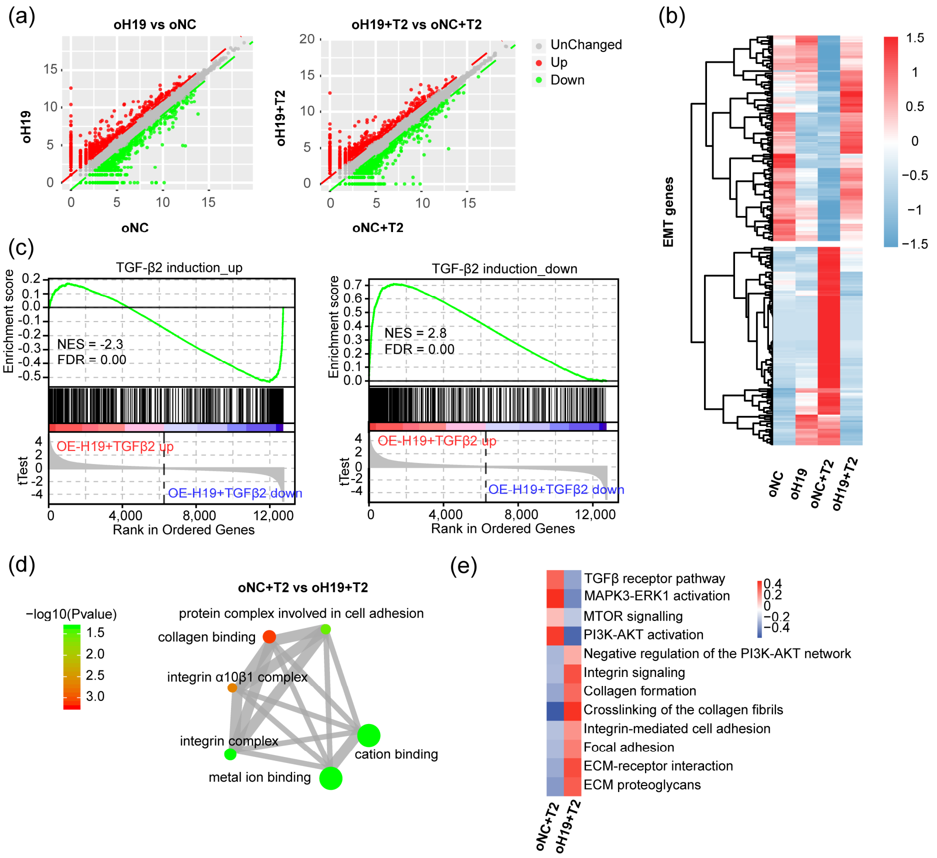

Figure S4. Transcriptomic analyses of the role of *H19* in regulating TGF-β2-induced EMT in lens epithelial cells.

(a) Scatter plots displayed all differentially expressed genes (DEGs) with statistical significance. Red indicated upregulated genes; green indicated downregulated genes. (b) Heatmaps illustrated downregulated (top) and upregulated (bottom) genes among different groups. (c) Gene Set Enrichment Analysis (GSEA) plotted gene signatures increased (left) and reduced (right) by TGF-β2 induction in *H19*-overexpressed LECs compared to control (o*H19*+T2 vs. oNC+T2). NES, normalized enrichment score: Positive NES indicated higher expression in *H19*-overexpressed LECs with TGF-β2 treatment (o*H19* +T2); negative NES suggested lower, instead. FDR, false discovery rate. (d) The bubble network showed top Gene Ontology(GO) terms of DEGs. The size of the bubbles represents the number of genes enriched in each GO term. The color intensity stands for statistical significance. (e) Key signaling pathways were uncovered in *H19*-overexpressed LECs compared to control (o*H19*+T2 vs. oNC+T2).

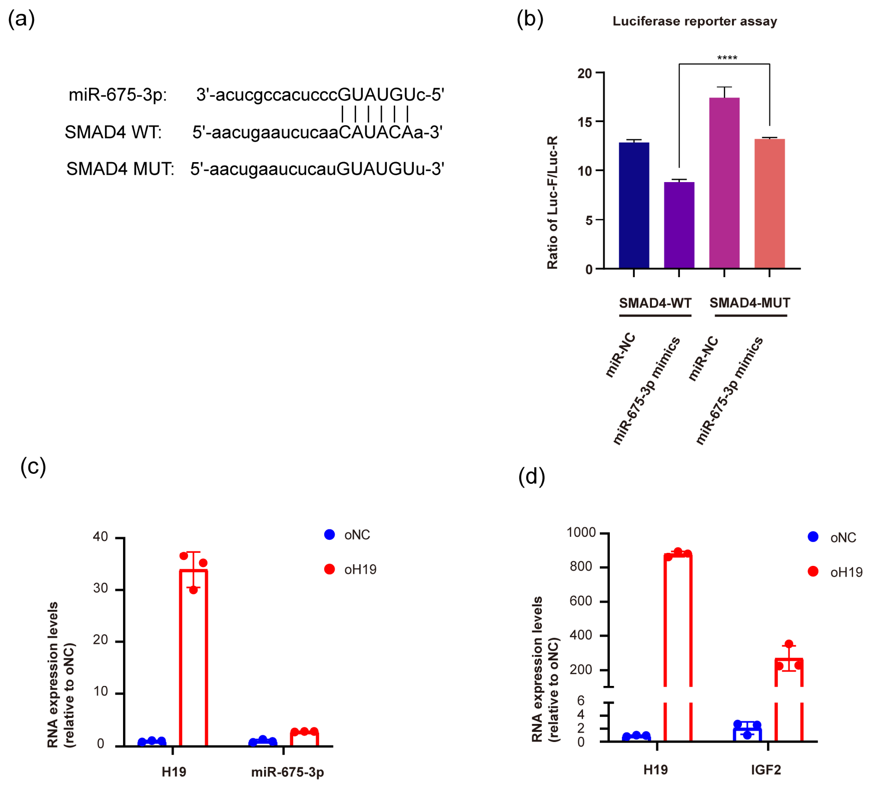

Figure S5. Validation of the potential *H19*/*miR675-3p*/*Smad4* axis and temporary exclusion of imprinting effects.

(a-b) Dual-luciferase reporter assay in SRA01/04 cells, after cotransfection with *miR-675-3p* mimics or miRNA negative control (miR-NC), combined with luciferase reporter plasmids incorporated with *Smad4* 3'-UTR (untranslated region) sequences or with mutated putative binding sites. (c) RNA analysis of *H19* and *miR-673-3p*, when *H19* was overexpressed in SRA01/04 cells. (d) RNA analysis of *H19* and IGF2(insulin growth factor 2) mRNA levels, when *H19* was upregulated in SRA01/04 cells.

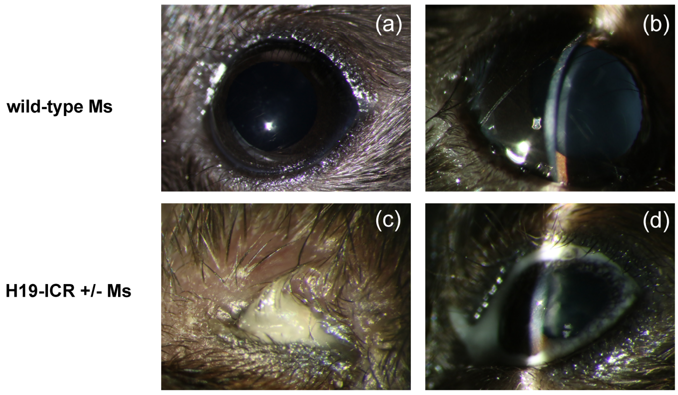

Figure S6. *H19*-ICR heterozygote mice displayed lens dysmorphogenesis or aplasia.
(a-d) Photos taken under slit lamps after the mice were anesthetized and their pupils were dilated. The ocular lenses of 3-week *H19*-ICR(imprinting control region) heterozygous knockout mice (c-d) and the wild-type littermates (a-b) were examined *in-vivo* by Haag-Streit BX 900 slit lamp with a diffuseillumination mode (a, c-OS), a narrow-slit mode (b, d-OD). OS, left eye; OD, right eye. Representative photos were shown here.
